# Photo-, osmo- and phonophobia in the premonitory phase of migraine: mistaking symptoms for triggers?

**DOI:** 10.1186/s10194-015-0495-7

**Published:** 2015-02-15

**Authors:** Laura H Schulte, Tim P Jürgens, Arne May

**Affiliations:** Department of Systems Neuroscience, University Medical Center Hamburg-Eppendorf, Martinistr. 52, Hamburg, D-22046 Germany

**Keywords:** Migraine, Tipping point, Premonitory symptoms, Triggers, Photophobia, Phonophobia, Nausea

## Abstract

**Background:**

Certain environmental stimuli are frequently reported as typical triggers of migraine pain. Whether these so-called triggers are independent precipitators of migraine pain or mere symptoms of the premonitory phase of migraine remains to be elucidated.

**Methods:**

In this retrospective cohort study of 1010 migraine patients of a tertiary headache center we assessed the frequency of common trigger factors, premonitory symptoms and accompanying symptoms as well as basic headache characteristics and demographic data.

**Results:**

Premonitory symptoms with an onset of 2 or more hours prior to the headache were present in 38.9% of migraine patients, the most frequent being a tense neck, phonophobia and difficulty concentrating. There was a clear overlap of certain trigger factors and the presence of corresponding premonitory symptoms: flickering or bright light as a trigger was associated with higher frequency of photophobia in the premonitory phase. The same applied to the presence of food craving and osmophobia in the premonitory phase and certain foods or odours as trigger factors.

**Conclusions:**

Our data thus support the view that commonly reported trigger factors of migraine are not so much independent precipitators of migraine pain, but that they are most likely just misinterpreted results of enhanced attention to certain stimuli mediated by typical premonitory symptoms of migraine pain.

## Background

Flickering light, bright sunlight, certain odours or certain foods are - among others - frequently reported as typical triggers of migraine pain [1-11]. However, empirical evidence that these factors can actually provoke migraine attacks is scarce [12,13], and it is currently not clear whether these factors are actual independent triggers of migraine pain, i.e. factors that at any given moment reliably precipitate migraine pain, or whether the increased intake of certain foods, the pronounced perception of e.g. certain lights or odours as adverse and even pain inducing might rather be a result of a slight photo- or osmophobia or food craving already present in the premonitory phase of migraine. It is therefore possible that these factors are rather a symptom than a true trigger of migraine [14,15]. To further elucidate this problem we analyzed the data of 1010 migraine patients.

## Methods

In this retrospective cohort study, 1805 patients of a tertiary headache center (Headache Outpatient Department of the University Medical Center Hamburg-Eppendorf) upon first admission were presented with a variety of tests and custom questionnaires presented in electronic form between November 2009 and July 2014. During this first admission patients were seen and diagnosed by a trained headache specialist (TPJ, AM), following the IHS criteria. Patients with a diagnosis of migraine with or without aura were included in the study.

**Figure 1:**
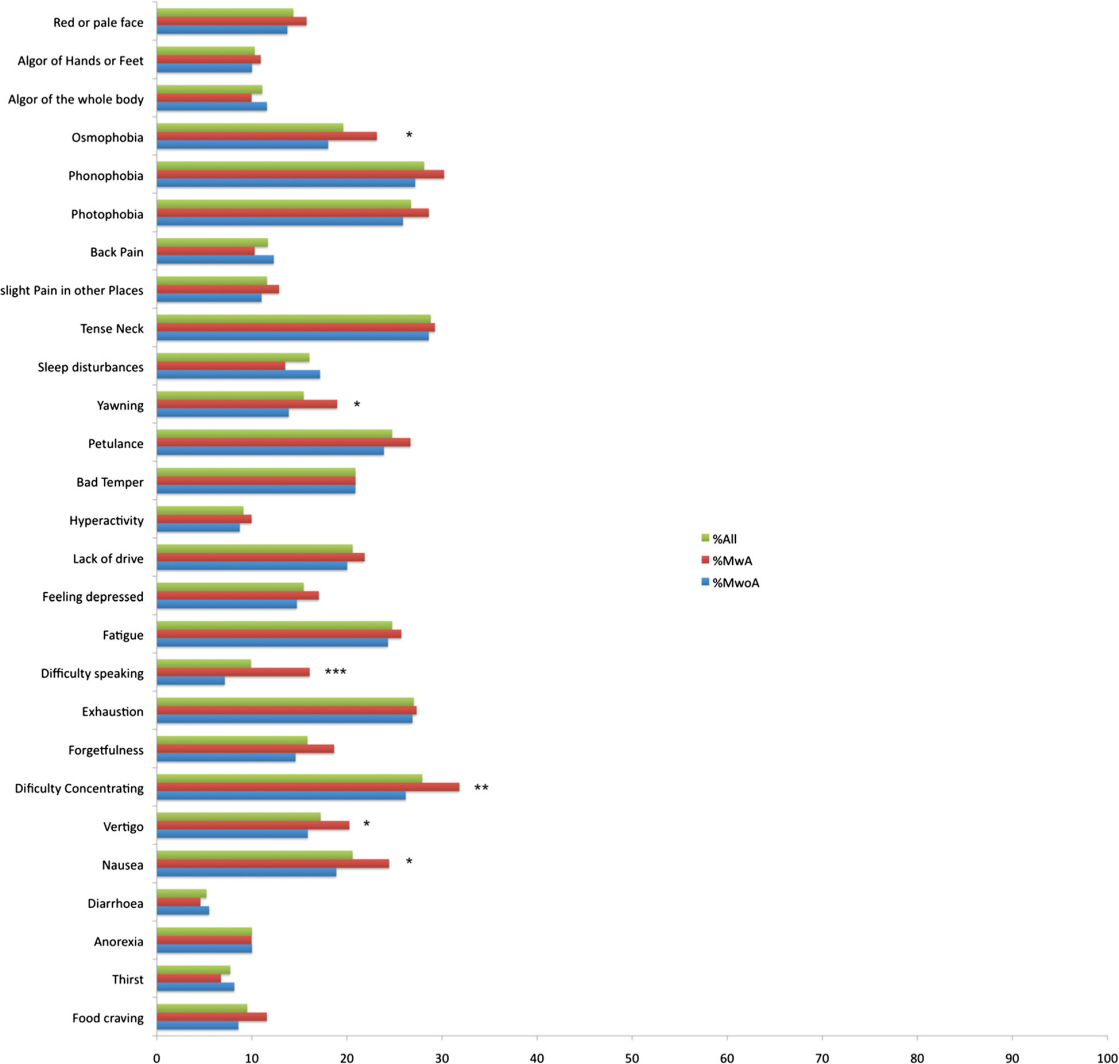
**Frequency of premonitory symptoms, shown as percentage of each of the three groups (all migraine patients, MwA, MwoA).** Symptoms were only counted as real premonitory symptoms, if patients reported an onset of 2 or more hours before the headache. Asterisks indicate significant differences between MwA and MwoA patients. *p < 0.05, **p < 0.01, ***p < 0.001. MwA = Migraine with Aura, MwoA = Migraine without Aura.

As part of the battery of questionnaires, patients were asked for the presence or absence of 27 typical migraine premonitory symptoms (see Figure 1). In one additional question the latency between onset of the reported premonitory symptoms and the headache phase was assessed. As we suspected a tendency among patients to not clearly differentiate between aura symptoms or predominant accompanying symptoms of the early pain phase of migraine and premonitory symptoms, we only regarded symptoms as truly premonitory, if patients reported an onset at least two hours prior to the headache. Only these patients were included when investigating the association of premonitory symptoms and trigger factors. Additionally we assessed basic headache characteristics, including 21 accompanying symptoms of the headache as well as 24 different trigger factors, among these flickering light, bright sunlight, certain foods and odours (see Table 1).

**Table 1:** **Headache triggers and accompanying symptoms of headache assessed by our questionnaires**

Headache triggers	Foods, stress, relieve from stress, weather/change in weather, bright sunlight, flickering light, heat, cold, physical exercise, sexual intercourse, irregular meals/skipping of meals, decreased water intake, sleep abundance, sleep deprivation, holiday, alcohol, smells/odours, resolvents, electric fields, medication, hormonal changes, specific head movements, other illnesses (e.g. common cold), touch
Accompanying symptoms of headache	Photophobia, phonophobia, osmophobia, need for rest, nausea, vomiting, allodynia of the skin, vertigo, facial rush, facial sweating, restlessness/agitation, swelling of lymph nodes, neck pain, conjunctival injection, tearing, eyelid oedema, ptosis, nasal congestion, rhinorrhoea, difficulty speaking, blurred vision

Data collection in a local data base and use of the data for scientific analysis and publication was approved by the local ethics committee of the chamber of physicians of Hamburg, Germany (submission number PV3185). Informed consent was obtained from all participants. Data were analyzed using SPSS Statistics. Categorical variables were compared using chi-square-test or – in case of cell numbers smaller than 6 – Fisher’s exact test. Continuous variables were analyzed using student’s *t* test or one way ANOVAs (in case of more than two groups). As the amount of missing data sets was low (<1.5%), we did not apply a special correction technique.

## Results

Of the 1805 headache patients presented with the questionnaires, 1010 patients were diagnosed with migraine. Of these, 699 had migraine without aura and 311 had migraine with aura (see Table 2 for demographic details of the study population). 389 migraine patients (resp. 38.5% of all migraine patients) reported premonitory symptoms starting at least 2 hours before the pain phase of migraine with no significant difference between MwoA and MwA (269 MwoA (38.5%) and 120 MwA (38.6%)). The 389 patients experiencing these phenomena reported an average of approximately 12 different premonitory symptoms with a mean onset of about 6.3 hours before the headache, the most frequent being a tense neck, phonophobia and difficulty concentrating (see Figure 1 for more detail). There were significant differences regarding the frequency of certain premonitory symptoms: Speaking difficulties, yawning, vertigo, nausea, osmophobia and difficulties concentrating were found significantly more often in MwA patients. There were significant but overall weak correlations between the sum of premonitory symptoms and age, sex, and MIDAS-Score.

**Table 2:** **Demographic characteristics of the study population**

	**MwoA**	**MwA**	**Total**
Number of patients	699	311	1010
Age	39.2 y	40.2 y	39.5 y
Sex	130 m, 569 f	51 m, 260 f	181 m, 829 f
Headache days per month	11.3 days	9.9 days	10.9 days
Disease duration	12 y	14.4 y	12.7 y
MIDAS-Score (Average)	44.9	45.9	45.2

When analyzing the interrelationship of reported trigger factors and corresponding premonitory symptoms, we found that migraine patients suffering from photophobia in the premonitory phase of migraine pain reported flickering light or bright sunlight as a trigger factor significantly more often than patients not experiencing photophobia in the premonitory phase. Accordingly, odours as a migraine trigger were significantly more frequent among patients reporting osmophobia as a premonitory symptom. The same distribution applied to certain foods as a trigger and the presence of food craving in the premonitory phase (p < 0.0001 for all comparisons). Additionally, in patients reporting nausea, photo-, osmo- or phonophobia in the premonitory phase these symptoms were significantly more frequent as accompanying symptoms of migraine pain (p < 0.0001 for all comparisons).

## Discussion

In the current study the prevalence rate of premonitory symptoms among migraine patients was 38.5%. We found a huge overlap between certain so-called triggers and the respective corresponding premonitory and accompanying symptoms of migraine pain.

There are currently five larger scale studies investigating the frequency of premonitory symptoms in migraine which found percentages between 32.9% and 86.9% of migraine patients reporting premonitory symptoms [16-20]. This is a wide frequency range which can possibly be explained by the varying ways of data acquisition, the variable number of premonitory symptoms investigated and the different patient populations in the five studies. In contrast to these studies we only regarded symptoms with an onset of 2 hours and more prior to the headache as truly premonitory. The found prevalence rate in our study is thus located in the lower range of previously reported rates. The most frequent premonitory symptoms were a tense neck, phonophobia and difficulty concentrating and are thus in line with previous studies [17,18,20].

As data were assessed retrospectively, a recall-bias might have led to an underestimation of triggers and premonitory symptoms. As however migraine patients have the migraine pain as a recall anchor (e.g. memory aid) and often pay special attention to possible triggers and premonitors, this attribution bias could have resulted in an over-estimation of the described phenomena. Additionally patients of a tertiary headache center are most likely more seriously afflicted by their migraine disorder and the associated symptoms than the general population of migraine sufferers. It is thus possible that the described distribution of symptoms and triggers might not reflect the average of migraine sufferers.

Our data show a clear association of the presence of certain symptoms in the premonitory phase of migraine and certain trigger factors corresponding to these symptoms, e.g. photophobia as a premonitory symptom and flickering or bright light as a corresponding trigger factor. The pronounced perception of certain factors as triggers might thus be merely a result of the presence of corresponding symptoms in the premonitory phase of migraine. The presence of e.g. photophobia, osmophobia or food craving might result in a pronounced perception of intense visual stimuli such as bright or flickering light as especially adverse and even pain inducing or in an increased intake of certain foods and thus lead to the conclusion in patients and physicians, that these factors can actually trigger migraine pain. As there is currently no study showing a reliable precipitation of migraine pain by typical trigger factors [12,13], it seems reasonable to assume that so-called triggers are really just early symptoms of the migraine attack.

It is however also possible that these classic triggers are in fact able to precipitate migraine pain but not independently and at all times: There is currently a lot of evidence that certain parameters of neuronal function (such as cortical excitability, response to evoked potentials, BOLD-response to standardized stimuli, activity levels of different areas of the brain and brainstem) in migraine patients change during different stages of the migraine cycle [21-25]. One study even could show that the level of perceived stress increased during the four days preceding a migraine attack [26]. Moreover, for a certain factor to be perceived as a migraine trigger by patients, a close temporal relationship between exposure time and the onset of the pain phase is important. Thus it becomes quite likely, that commonly reported trigger factors are not independent precipitators of migraine pain, but that their ability to actually provoke attacks depends on the stage of the migraine cycle during which they are applied.

Our data further support the assumption that in some patients the typical accompanying symptoms of migraine pain actually precede the pain phase [1,17-20,27] and thus offer additional evidence for the common assumption that a migraine attack is not just an isolated event of pain but it is more likely a continuous up and down of certain sensory and bodily functions on top of which comes the headache. If migraine is understood as a threshold problem, any external stimulus may or may not be perceived as a trigger, depending on the threshold level. The threshold itself oscillates and as long as it is above an individual mark a so-called “trigger” has no consequences. Once the threshold level falls below that mark, the incoming sensation will be perceived as unpleasant and misinterpreted as a trigger. However, the change in threshold is the original mechanism behind the origination of an attack and the growing unpleasantness of a usually inert external stimulus is probably nothing more than the first perception of the decreasing threshold. It probably depends on the susceptibility of a given migrainous individual whether the pronounced and possibly unpleasant perception of light or smell or other stimuli are the first symptom of the attack and photophobia, osmophobia, nausea or phonophobia will then be one of the distinctive following symptoms in the attack.

## Conclusions

Our data corroborate the view that most of the reported common trigger factors of migraine are not so much independent triggers that at any time might precipitate an acute attack of migraine pain, but that they are most likely just misunderstood symptoms of the premonitory phase of migraine pain, which is already part of the migraine attack. Future studies investigating “migraine” and also “triggers” have to carefully assess the precise state of the migraine cycle, which must include not only the last but also the beginning of the next attack. We need to characterize the involved neuronal and autonomic subnetworks and their connections during all parts of the migraine cycle if we are ever to understand migraine.
